# Interactions between Caveolin-1 polymorphism and Plant-based dietary index on metabolic and inflammatory markers among women with obesity

**DOI:** 10.1038/s41598-022-12913-y

**Published:** 2022-05-31

**Authors:** Faezeh Abaj, Atieh Mirzababaei, Dorsa Hosseininasab, Niki Bahrampour, Cain C. T. Clark, Khadijeh Mirzaei

**Affiliations:** 1 Department of Community Nutrition, School of Nutritional Sciences and Dietetics, Tehran University of Medical Sciences (TUMS), P.O. Box: 14155-6117, Tehran, Iran; 2 Department of Nutrition, Science and Research Branch, Islamic Azad University, Tehran, Iran; 3 Department of Nutrition, Science and Research Branch, Islamic Azad University (SRBIAU), Tehran, Iran; 4 Centre for Intelligent Healthcare, Coventry University, Coventry, CV1 5FB UK

**Keywords:** Diseases, Genetics, Genetic interaction

## Abstract

A series of recent studies have indicated that the Caveolin-1 (CAV-1) gene variant may be associated with metabolic and inflammatory markers and anthropometric measures. Furthermore, it has been shown that a plant-based dietary index (PDI) can elicit a positive impact on these metabolic markers. Therefore, we sought to examine whether PDI intakes may affect the relationship between CAV-1 (rs3807992) and metabolic factors, as well as serum inflammatory markers and anthropometric measures, in women with obesity. This current study consisted of 400 women with overweight and obesity, with a mean (SD) age of 36.67 ± 9.10 years. PDI was calculated by a food frequency questionnaire (FFQ). The anthropometric measurements and serum profiles were measured by standard protocols. Genotyping of the CAV-1(rs3807992) was conducted by the PCR–RFLP method. The following genotypic frequencies were found among the participants: GG (47.8%), AG (22.3%), and AA (2.3%). In comparison to GG homozygotes, risk-allele carriers (AA + AG) with higher PDI intake had lower ALT (P: 0.03), hs-CRP (P: 0.008), insulin (P: 0.01) and MCP-1 (P: 0.04). Furthermore, A-allele carriers were characterized by lower serum ALT (P: 0.04), AST (P: 0.02), insulin (P: 0.03), and TGF-β (P: 0.001) when had the higher following a healthful PDI compared to GG homozygote. Besides, risk-allele carriers who consumed higher unhealthful PDI had higher WC (P: 0.04), TC/HDL (P: 0.04), MCP-1 (P: 0.03), and galactin-3 (P: 0.04). Our study revealed that A-allele carriers might be more sensitive to PDI composition compared to GG homozygotes. Following a healthful PDI in A-allele carriers may be associated with improvements in metabolic and inflammatory markers and anthropometric measures.

## Introduction

Obesity is defined by an excess in the accumulation of lipids in adipose tissue; this accumulation becomes detrimental when it happens in visceral fat^1^. Indeed, waist circumference (WC) (an indirect measure of visceral fat accumulation) is correlated with the progression of particular metabolic disorders containing cardiovascular diseases (CVDs) and type 2 diabetes (T2DM)^2^. The prevalence of obesity is universally higher in women^3^; additionally, women with high body mass index (BMI) are considered at greater risk of T2DM^4^, atherosclerotic cardiovascular disease^5^, hypertension^6^, dyslipidemia^7^, and endocrine disorders^8^. Moreover, increased levels of hepatic enzymes, such as alanine aminotransferase (ALT) and aspartate aminotransferase (AST), are common in obesity and their prevalence increases gradually with increasing BMI^9^. Numerous population studies show that low-grade and chronic inflammation is often augmented in patients with obeisty^10^. This is characterized by incremented circulating levels of pro-inflammatory cytokines—particularly Interleukin-6 (IL- 6)—and of the monocyte chemoattractant protein (MCP-1), both generated by the adipose tissue^11–14^.

Diet is one of the most notable variables that have life-long effects on obesity and metabolic risk factors^15^. There is abundant evidence to suggest that dietary patterns are related to inflammatory mediators and obesity as an inflammatory-related disease^16–19^; indeed, a review article demonstrated that a plant-based diet (PBD) could alleviate certain inflammatory markers, such as IL-6^20^. This kind of dietary pattern has been described as possessing a high intake of vegetables, fruits, grains, and legumes, which could lead to reductions in both inflammation and obesity^21^. Also, dietary inflammatory index, in which the consumption of food items that can increase body inflammations, such as saturated fatty acid, trans fatty acids, and refined grains, were positively associated with fat mass and concentration of MCP-1^22^. Several dietary intervention studies have shown that healthy plant foods, such as flavonoids, can modulate inflammatory cytokines^23–25^. Therefore, PBD may be associated with an improvement in obesity-related inflammatory profiles and inhibition of chronic diseases risks. Indeed, recent studies have revealed that there might be an interaction between specific genes such as Caveolin-1 and particular dietary patterns^26,27^.

Caveolin-1 (CAV-1), is the major structural protein of caveolae, which is located on human chromosome 7 (7q31.1) and it includes 3 exons that select intronic single nucleotide polymorphisms (SNPs). CAV-1 works in a scaffolding capacity and has been involved in transmembrane signaling^28^. CAV-1 is a vital constituent of the lipid raft that controls their activity and cooperates with numerous signaling pathways, including steroid receptors^29^. Moreover, CAV-1 gene variants have been shown to be correlated with insulin resistance (IR), diabetes mellitus, dyslipidemia, and metabolic syndrome^30^. Based on previous studies, the adipocyte seems to have higher concentrations of caveolae than any other cell^31,32^. Additionally, some studies have shown that diets containing foods with antioxidant features such as PBD, can modulate CAV-1 levels and caveolin function for the debarment of chronic diseases^33–36^.

Given the rising prevalence of obesity and the lack of studies performed on Caveolin-1 (rs3807992) and plant-based dietary index (PDI), as well as its association with metabolic and inflammatory markers among women, the present study sought to evaluate the interactions between Caveolin-1 (rs3807992) and PDI on metabolic and inflammatory markers in Iranin women with overweight and obesity.

## Methods

### Study population

This cross-sectional study, including 400 women with obesity, was conducted in the health care center of Tehran, Iran. The subjects were chosen via a random cluster random sampling method, where the inclusion criteria were: women with overweight (BMI ≥ 25 kg/m^2^) or obesity (BMI ≥ 30 kg/m^2^), aged above 18 years, before menopause, not being pregnant or lactating and smoker. The women who had any chronic disease (T2DM), CVDs, polycystic ovary syndrome (PCOS), non-alcoholic fatty liver disease, etc.), taking any supplements or medications (Atorvastatin, Cholestyramine, etc.), and weight loss program were excluded. The general characteristics of the participants were collected via a demographic questionnaire. The study protocol was approved by the Ethics Commission of Tehran University of Medical Sciences (IR.TUMS.VCR.REC. 97-03-161-41017)^37^. Total calorie intake was accepted in the range between 800 and 4200 kcal per day, with intakes outside of this range leading to exclusion from the study^38^. All of the participants completed a written informed consent form before taking part in the study.

### General, anthropometric and physical activity assessments

Height was measured using Seca 216 to the nearest 0.1 cm and weight of subjects was also assessed using Seca scale to the nearest 0.1 kg, with light clothes and unshod, in a standing position. WC was measured at the point of the smallest girth by a trained expert. BMI was calculated as weight/(height)^2^. In addition, other anthropometric variables were measured using a multi-frequency bioelectrical impedance analyzer InBody 770 scanner (Inbody Co., Seoul, Korea), with participants wearing light clothes without any metal subjects^39^. In addition, physical activity (PA) was assessed via the validated International Physical Activity Questionnaire^40^. All participants' blood pressure was monitored using an automatic sphygmomanometer according to established procedures (OMRON, Germany).

### Dietary assessments and plant-based dietary intake

A validated 147-items semi-quantitative food frequency questionnaire (FFQ) was used by an expert to assess the dietary intake of the subjects. Grams of foods and beverages were entered into NUTRITIONIST IV software (version 7.0; N-Squared Computing, Salem, OR)^41,42^. According to the plant-based dietary intake, which was divided to overall plant-based diet index (PDI), healthy PDI (hPDI), and unhealthy PDI (uPDI), the intakes of the subjects were divided to 18 groups, which were: animal (Dairy, animal fat, egg, meat, fish and seafood, and miscellaneous animal-based foods), healthy (whole grains, vegetables, fruits, legumes, nuts, vegetable oils, tea, and coffee), and unhealthy plant-based foods (fruit juices, sugar-sweetened beverages, refined grains, sweets and desserts, and potatoes)^43,44^. Foods were scored 1–10, after they were transformed to deciles. PDI, hPDI, and uPDI were assessed as scoring 10 and 1 for the highest and lowest deciles of plant food intake, healthy plant foods, and unhealthy plant foods, respectively. Conversely, unhealthy plant foods and animal food items were scored 1 to 10 for the highest and lowest deciles of animal foods and unhealthy plants foods, respectively.

### Biochemical measurements

After 10–12 h night fasting, blood samples were gathered at the Nutrition and Biochemistry laboratory of the School of Nutritional Sciences and Dietetics, TUMS. Triacylglycerol kits (Pars Azmoon Inc, Tehran, Iran) were used to measure serum triglycerides (TG) by colorimetric method tests with Glycerol-3-phosphate oxidase Phenol 4-Aminoantipyrine Peroxidase (GPO-PAP). The cholesterol oxidase Phenol 4-Aminoantipyrine Peroxidase (CHOD-PAP) was used to calculate total cholesterol, and the direct method and immunoinhibition were used to measure low-density lipoprotein (LDL) and high-density lipoprotein (HDL). Pars Azmoon (Pars Azmoon Inc., Tehran, Iran) provided all of the kits. ALT, AST, high-sensitivity C-reactive protein (hs-CRP), MCP-1, interleukin 1 beta (IL-1β), and transforming growth factor-beta (TGFβ) were measured via standard protocols. Plasminogen activator inhibitor-1 (PAI-1) (Human PAI-1*96 T ELISA kit Crystal Company) was measured in triplicate. TC was divided into HDL to discern the ratio of TC/HDL, in addition, LDL was divided by HDL to discern the ratio of LDL/HDL. Serum insulin level was also measured by radioimmune assay. More detailed about biochemical measuring are reported in our previous study^45^.

### DNA analysis

Based on a previous study^27^, the Mini Columns kit (Type G; Genall; Exgene) was used for DNA extraction. The CAV-1 SNP (rs3807992) was genotyped by PCR–RFLP method, using primers, Forward: 3′AGTATTGACCTGATTTGCCATG5′ Reverse: 5′GTCTTCTGGAAAAAGCACATGA-3′. The sequencing process was performed using the ABI PRISM 3730 automated sequencer (Applied Biosystems, Foster City, Calif, USA)^27^.

### Statistical analyses

The Kolmogorov–Smirnov test was used for assessing the normality of the data. The Hardy–Weinberg equilibrium and comparison of categorical variables were assessed with the x^2^ test. PDI was transformed to tertiles based on the trends. Quantitative (mean ± SD) and qualitative variables (n (%)) were evaluated via one-way analysis of variance (ANOVA) and chi-square among PDI tertiles, respectively. Genotype groups were considered as a dominant inherent model (AA + AG) versus GG homozygous. Analysis of covariance (ANCOVA) was performed for the adjustment model (adjusting for age, physical activity and energy intake and diastolic blood pressure (DBP)). A generalized linear regression model (GLZM) was used to analyze the interactions between CAV-1 polymorphism (rs3807992) and PDI. Data were analyzed using Statistical Package for Social Sciences (SPSS Inc., Chicago, IL, version 25) and a P-value < 0.05 was considered as significant; but for interactions, P < 0.1 was considered significant.

### Ethics approval and consent to participate

All methods were performed in accordance with the relevant guidelines and regulations. The protocol of the study was approved by the ethics committee of TUMS (Ethics number: 97-03-161-41017). All participants completed a written informed consent.

## Results

### Study population characteristics

The participants in this study consisted of 400 women, with a mean (SD) age of 36.67 ± 9.10 years. The majority of individuals (72.4%) were married, had a university education (47.8%), and had a history of obesity (71.2 percent). The following genotypic frequencies were found among the participants: GG (47.8%), AG (22.3%), and AA (2.3%).

### The difference in means of biochemical parameters and body composition between *CAV-1 rs3807992* genotypes

The comparison of variables including anthropometrics and biochemical parameters according to two genotypes groups (GG and AG + AA) is given in Table 1. The results of this study revealed that DBP (P = 0.007), body fat mass (BFM) (P = 0.01), and fat mass index (FMI) (P = 0.009) were higher in participants carrying the A allele, compared with individuals in the GG genotype. Moreover, A-allele carriers had significantly lower serum HDL (P < 0.001) and LDL (P = 0.01) compared to GG homozygotes; although, the TC/HDL (P = 0.008) ratio was significantly higher. Furthermore, there was no significant association between this polymorphism and other biochemical parameters including TG, inflammatory markers (hs-CRP, MCP-1, TGFβ), and liver enzymes (ALT and AST) in both crude and adjusted models (P > 0.05) (Table 1).

**Table 1:** Characteristics of the study population across rs 3807992 genotypes.

Variables	Genotypes		
	GG (n = 207)	AG + AA (n = 193)	P-value
**Demographic variables**			
Age (years)	37.56 ± 9.49	35.75 ± 8.78	0.05
Physical activity (MET-min/week)	1215.46 ± 2033.81	1199.02 ± 2251.97	0.42
**Blood parameters**			
FBS (mg/dl)	87.98 ± 9.62	86.95 ± 9.75	0.40
Insulin (mlU/ml)	1.21 ± 0.22	1.22 ± 0.25	0.61
HOMA-IR	3.27 ± 1.21	3.41 ± 1.36	0.40
TC (mg/dl)	186.76 ± 33.74	182.71 ± 37.36	0.37
TG (mg/dl)	113.11 ± 51.20	125.08 ± 66.66	0.11
HDL (mg/dl)	49.07 ± 11.16	44.04 ± 10.16	** < 0.001**
LDL (mg/dl)	98.88 ± 22.66	91.27 ± 25.07	**0.01**
TC/HDL	3.93 ± 0.89	4.43 ± 1.91	**0.008**
LDL/HDL	2.07 ± 0.53	2.14 ± 0.64	0.35
Hs-CRP	4.18 ± 4.40	4.33 ± 4.76	0.79
ALT	19.04 ± 13.95	20.14 ± 14.19	0.53
AST	18.27 ± 7.44	17.95 ± 8.29	0.75
MCP-1	51.49 ± 102.26	48.42 ± 81.14	0.80
TGF-β	54.47 ± 16.18	55.34 ± 19.72	0.12
Galactin-3	4.28 ± 8.16	3.47 ± 5.56	0.60
**Blood pressure**			
SBP (mmHg)	110.31 ± 12.63	112.90 ± 14.75	0.12
DBP (mmHg)	75.87 ± 10.77	79.31 ± 10.06	**0.007**
**Anthropometric parameters**			
Weight (kg)	79.26 ± 10.35	80.90 ± 11.68	0.15
Height (cm)	161.30 ± 6.08	160.96 ± 5.58	0.56
BMI (kg/m^2^)	30.57 ± 3.88	31.30 ± 3.93	0.07
WC (cm)	98.22 ± 9.30	99.87 ± 9.49	0.08
WHR	0.93 ± 0.05	1.41 ± 6.58	0.30
**Body composition**			
SMM (kg)	25.34 ± 3.27	25.63 ± 3.55	0.40
FFM (kg)	46.26 ± 5.48	46.54 ± 5.83	0.62
BFM (kg)	33.49 ± 7.96	35.64 ± 9.15	**0.01**
FMI	12.93 ± 3.25	13.83 ± 3.46	**0.009**
BF (%)	41.62 ± 5.47	42.68 ± 5.50	0.05
VFA (cm)	170.42 ± 25.32	176.65 ± 39.24	0.98

Variables are presented as mean ± SD for continuous variables.

*FBS* fasting blood sugar, *HOMA-IR* homeostatic model assessment for insulin resistance, *TC* total cholesterol, *TG* triglyceride, *HDL* high density lipoprotein, *LDL* low density lipoprotein, *SBP* systolic blood pressure, *DBP* diastolic blood pressure, *BMI* body mass index, *SMM* skeletal muscle mass, *FFM* fat free mass, *FMI* fat mass index, *BFM* body fat mass, *BFP* body fat percentage, *WHR* waist hip ratio, *WC* waist circumference, *VFA* visceral fat area, *AST* aspartate aminotransferase, *ALT* alanine aminotransferase, *MCP-1* monocyte chemoattractant protein-1, *TGFβ* Transforming growth factor beta.

Significant values are in bold.

P-value is found by Independent T test.

### The difference in means of biochemical parameters and body composition between PDI, hPDI and uPDI

The result of comparison of biochemical parameters and body composition between PDI, hPDI and uPDI are shown in Tables 2, 3 and 4. These results displayed that women with higher PDI were older (P = 0.03) and had higher physical activity (P < 0.001) than those with lower PDI. Women with higher healthful PDI had significantly lower LDL/HDL ratios in both crude (P = 0.04) and adjusted models (P = 0.03); although there was no statistically significant difference (P > 0.05) from the low to high unhealthful PDI.

**Table 2:** Description of characteristics among intake of PDI.

Variables		PDI groups			
		Low adherence (n = 158)	High adherence (n = 130)	P-value	P-value*
**Demographic variables**					
Age (years)		35.98 ± 8.88^1^	37.29 ± 7.93	0.19	0.21
Physical activity (MET-min/week)		1095.89 ± 1988.02	1342.57 ± 2253.62	0.35	0.33
**Blood parameters**					
FBS (mg/dl)		87.43 ± 9.62	87.59 ± 9.71	0.90	0.72
Insulin (lU/ml)		1.21 ± 0.20	1.21 ± 0.25	0.91	0.68
HOMA-IR		3.40 ± 1.27	3.24 ± 1.28	0.34	0.57
TC (mg/dl)		184.33 ± 37.98	185.67 ± 33.85	0.77	0.69
TG (mg/dl)		117.51 ± 60.55	118.54 ± 58.81	0.89	0.75
HDL (mg/dl)		47.27 ± 11.02	46.13 ± 10.66	0.41	0.92
LDL (mg/dl)		95.16 ± 25.06	94.51 ± 22.99	0.83	0.91
TC/HDL		4.09 ± 1.61	4.24 ± 1.37	0.43	0.89
LDL/HDL		2.06 ± 0.55	2.13 ± 0.62	0.34	0.78
**Blood pressure**					
SBP (mmHg)		109.88 ± 12.11	113.84 ± 15.35	**0.01**	0.09
DBP (mmHg)		77.09 ± 8.40	78.55 ± 10.92	**0.02**	0.38
**Anthropometric parameters**					
Weight (kg)		79.01 ± 10.04	80.38 ± 11.24	0.28	0.74
Height (cm)		161.17 ± 6.17	161.39 ± 5.64	0.74	0.51
BMI (kg/m^2^)		30.47 ± 3.79	30.99 ± 3.75	0.24	0.47
WC (cm)		97.97 ± 9.07	99.21 ± 9.78	0.26	0.51
WHR		1.50 ± 7.22	0.93 ± 0.05	0.37	0.58
**Body composition**					
SMM (kg)		25.43 ± 3.06	25.97 ± 3.57	0.16	0.94
FFM (kg)		46.34 ± 5.17	47.29 ± 6.03	0.15	0.99
BFM (kg)		33.17 ± 8.02	35.06 ± 9.36	0.06	0.37
BF (%)		41.27 ± 5.47	41.83 ± 5.63	0.39	0.33
VFA (cm)		170.21 ± 36.72	166.07 ± 41.40	0.73	0.55
RMR (kcal)		1565.22 ± 246.63	1589.23 ± 274.73	0.43	0.44
**Qualitative variables**					
Marriage status	Single	36 (22.6%)	19 (14.7%)	0.11	0.12
Occupation	Unemployed	80 (51%)	92(71.3%)	**0.002**	**0.002**
Education	illiterate	2 (1.3%)	1(0.8%)	**0.01**	**0.01**
	Diploma	56 (35.2%)	70 (43.9%)		
	University	101 (63.6%)	159(55.2%)		
Weight loss history in past year	No	75 (47.5%)	58 (46.4%)	0.90	0.88

Variables are presented as mean ± SD for continuous variables and frequency for categorical variables.

*PDI* plant-based diet index, *FBS* fasting blood sugar, *HOMA-IR* homeostatic model assessment for insulin resistance, *TC* total cholesterol, *TG* triglyceride, *HDL* high density lipoprotein, *LDL* low density lipoprotein, *SBP* systolic blood pressure, *DBP* diastolic blood pressure, *BMI* body mass index, *SMM* skeletal muscle mass, *FFM* fat free mass, *BFM* body fat mass, *BF* body fat percentage, *WHR* waist hip ratio, *WC* waist circumference, *VFA* visceral fat area, *RMR* resting metabolic rate.

Significant values are in bold.

P values from t test for continuous variables and chi-square test for categorical variables.

*P-value as found by ANCOVA, and adjusted for (age, physical activity and energy intake and diastolic blood pressure (DBP)).

**Table 3:** Description of characteristics among intake of hPDI.

Variables		hPDI groups			
		Low adherence (n = 158)	High adherence (n = 130)	P-value	P-value*
**Demographic variables**					
Age (years)		35.57 ± 8.81^1^	37.65 ± 7.97	**0.03**	**0.02**
Physical activity (MET-minutes/week)		893 ± 849.77	1527.65 ± 2853.94	** < 0.001**	** < 0.001**
**Blood parameters**					
FBS (mg/dl)		87.90 ± 9.70	87.11 ± 9.61	0.52	0.75
Insulin (lU/ml)		1.22 ± 0.24	1.20 ± 0.21	0.35	0.89
HOMA-IR		3.46 ± 1.37	3.20 ± 1.16	0.12	0.92
TC (mg/dl)		181.61 ± 38.26	188.21 ± 33.77	0.15	0.66
TG (mg/dl)		118.13 ± 63.41	117.80 ± 55.90	0.96	0.36
HDL (mg/dl)		47.13 ± 10.55	46.41 ± 11.18	0.60	0.85
LDL (mg/dl)		92.23 ± 25.01	97.50 ± 23.01	0.08	0.93
TC/HDL		4.09 ± 1.81	4.23 ± 1.14	0.48	0.20
LDL/HDL		2.16 ± 0.55	2.02 ± 0.61	**0.04**	**0.03**
**Blood pressure**					
SBP (mmHg)		111.93 ± 14.31	111.35 ± 13.20	0.72	0.56
DBP (mmHg)		77.38 ± 9.64	78.14 ± 9.62	0.51	0.50
**Anthropometric parameters**					
Weight (kg)		79.77 ± 10.06	79.45 ± 11.17	0.80	0.86
Height (cm)		161.82 ± 5.96	160.65 ± 5.85	0.09	0.76
BMI (kg/m^2^)		30.61 ± 3.85	30.79 ± 3.71	0.69	
WC (cm)		98.95 ± 9.42	98.06 ± 9.38	0.42	0.89
WHR		1.53 ± 7.41	0.92 ± 0.05	0.33	0.34
**Body composition**					
SMM (kg)		25.96 ± 3.14	25.36 ± 3.46	0.12	0.59
FFM (kg)		47.30 ± 5.35	46.19 ± 5.80	0.09	0.51
BFM (kg)		34.24 ± 9.29	33.78 ± 8.01	0.65	0.55
BF (%)		41.22 ± 5.98	41.86 ± 5.01	0.32	0.23
VFA (cm)		174.69 ± 40.52	161.41 ± 38.28	0.28	0.24
**Qualitative variables**					
Marriage status	Single	34(22.5%)	21 (15.3%)	0.18	0.22
Occupation	Unemployed	94(63.1%)	78(56.9%)	0.09	0.08
Education	illiterate	2 (1.3%)	1(0.7%)	0.38	0.35
	Diploma				
	University	86 (56.9%)	73 (53.3%)		
Weight loss history in past year	No	71 (46.7%)	62 (47.3%)	0.91	0.92

Variables are presented as mean ± SD for continuous variables and frequency for categorical variables.

*hPDI* healthy plant-based diet index, *FBS* fasting blood sugar, *HOMA-IR* homeostatic model assessment for insulin resistance, *TC* total cholesterol, *TG* triglyceride, *HDL* high density lipoprotein, *LDL* low density lipoprotein, *SBP* systolic blood pressure, *DBP* diastolic blood pressure, *BMI* body mass index, *SMM* skeletal muscle mass, *FFM* fat free mass, *BFM* body fat mass, *BF* body fat percentage, *WHR* waist hip ratio, *WC* waist circumference, *VFA* visceral fat area, *RMR* resting metabolic rate.

Significant values are in bold.

P values from t test for continuous variables and chi-square test for categorical variables.

*P-value as found by ANCOVA, and adjusted for (age, physical activity and energy intake and diastolic blood pressure (DBP)).

**Table 4:** Description of characteristics among intake of uPDI.

Variables		uPDI groups			
		Low adherence (n = 157)	High adherence (n = 131)	P-value	P-value*
**Demographic variables**					
Age (years)		36.87 ± 8.61	36.21 ± 8.32	0.51	0.52
Physical activity (MET-min/week)		1300.12 ± 1992.97	1092.14 ± 2237.48	0.92	0.88
**Blood parameters**					
FBS (mg/dl)		87.18 ± 8.68	87.90 ± 10.76	0.56	0.35
Insulin (lU/ml)		1.22 ± 0.22	1.20 ± 0.23	0.47	0.34
HOMA-IR		3.21 ± 1.17	3.47 ± 1.39	0.12	0.14
TC (mg/dl)		185.16 ± 35.72	184.62 ± 36.85	0.90	0.99
TG (mg/dl)		119.06 ± 61.60	116.57 ± 57.41	0.74	0.80
HDL (mg/dl)		47.33 ± 11.04	46.07 ± 10.62	0.36	0.42
LDL (mg/dl)		96.40 ± 23.72	92.98 ± 24.59	0.27	0.33
TC/HDL		4.06 ± 1.06	4.28 ± 1.92	0.26	0.25
LDL/HDL		2.09 ± 0.55	2.09 ± 0.63	0.95	0.99
Blood pressure					
SBP (mmHg)		111.67 ± 13.52	111.64 ± 14.13	0.98	0.84
DBP (mmHg)		77.15 ± 9.27	78.45 ± 10.01	0.26	0.20
**Anthropometric parameters**					
Weight (kg)		80.46 ± 10.74	78.61 ± 10.37	0.39	0.2
Height (cm)		161.40 ± 5.57	161.11 ± 6.35	0.10	0.84
BMI (kg/m^2^)		30.93 ± 3.83	30.43 ± 3.70	0.27	0.3
WC (cm)		99.22 ± 9.56	97.69 ± 9.16	0.17	0.18
WHR		0.93 ± 0.05	1.62 ± 7.95	0.28	0.34
**Body composition**					
SMM (kg)		25.93 ± 3.27	25.37 ± 3.33	0.15	0.26
FFM (kg)		47.17 ± 5.52	46.29 ± 5.64	0.18	0.32
BFM (kg)		34.49 ± 9.04	33.44 ± 8.24	0.30	0.45
BF (%)		41.54 ± 5.75	41.51 ± 5.30	0.96	0.99
VFA (cm)		165.32 ± 40.19	172 ± 49.84	0.59	0.54
RMR (kcal)		1574.26 ± 259.60	1577.93 ± 260.11	0.90	0.88
**Qualitative variables**					
Marriage status	Single	26(16.5%)	29 (22.3%)	0.36	0.41
Occupation	Unemployed	97(61.8%)	77(59.7%)	0.37	0.35
Education	illiterate	1 (0.6%)	2(1.5%)	0.41	0.42
	Diploma				
	University				
Weight loss history in past year	No	67 (43.8%)	66 (50.8%)	0.28	0.33

Variables are presented as mean ± SD for continuous variables and frequency for categorical variables.

*UhPDI* healthy plant-based diet index, *FBS* fasting blood sugar, *HOMA-IR* homeostatic model assessment for insulin resistance, *TC* total cholesterol, *TG* triglyceride, *HDL* high density lipoprotein, *LDL* low density lipoprotein, *SBP* systolic blood pressure, *DBP* diastolic blood pressure, *BMI* body mass index, *SMM* skeletal muscle mass, *FFM* fat free mass, *BFM* body fat mass, *BF* body fat percentage, *WHR* waist hip ratio, *WC* waist circumference, *VFA* visceral fat area, *RMR* resting metabolic rate.

Significant values are in bold.

P values from t test for continuous variables and chi-square test for categorical variables.

*P-value as found by ANCOVA, and adjusted for (age, physical activity and energy intake and diastolic blood pressure (DBP)).

### Interactions between PDIs and *CAV-1 rs3807992* genotypes on metabolic and inflammatory markers and anthropometric measures

The interaction effects between CAV-1 variants at rs3807992 and PDIs (healthful and unhealthful) on metabolic and inflammatory markers, including TC, HDL, LDL, TC, TC/HDL, LDL/HDL, TG, ALT, AST, hs-CRP, MCP-1, PAI-1, and IL-1β, are shown in Tables 5 and 6.

**Table 5:** Interactions between CAV-1 rs3807992 and PDI on metabolic and inflammatory markers and anthropometric measures.

	β (95%CI) (AA + AG) 1(Ref) GG			P**
	Crude	P*	Adjusted	
WC (cm)	− 0.20 (− 5.35, 2.45)	0.80	− 0.13 (− 4.56, 4.30)	0.95
TC (mg/dl)	− 5.98 (− 24.3,12.33)	0.52	− 6.57 (− 24.76, 11.61)	0.47
HDL-C (mg/dl)	4.20 (− 1.24, 9.64)	0.13	4.48 (− 1.06, 10.02)	0.11
LDL-C (mg/dl)	0.79 (− 11.33,12.92)	0.89	− 0.28 (− 12.27, 11.71)	0.96
TC/HDL	− 0.49 (− 1.25,0.27)	0.20	− 0.05 (− 0.11,0.005)	0.07
ALT(IU L)	− 7.11 (− 13.81,− 0.4)	**0.03**	− 7.51 (− 14.45, − 0.57)	**0.03**
AST (IU L)	− 2.85 (− 6.65,0.95)	**0.14**	− 3.13 (− 7.07, 0.81)	**0.12**
Insulin (pmol/L)	− 0.13 (− 0.24, − 0.01)	**0.03**	− 0.14 (− 0.26,− 0.02)	**0.01**
hs-CRP (mg/L)	− 2.89 (− 5.27, − 0.51)	**0.01**	− 3.30 (− 5.76, − 0.85)	**0.008**
MCP-1(ng/ml)	− 45.75 (− 96.3,4.48)	**0.07**	− 54.51(− 107.72, − 1.30)	**0.04**
TGFβ (ng/ml)	− 142.73 (− 365.94, 80.47)	0.21	− 278.06 (− 572.39, 16.26)	0.06
Galactin 3 (ng/ml)	− 0.11 (− 6.42, 6.20)	0.97	− 2.28 (− 9.57, 5.03)	0.53

Values are represented as β (95%CI).

*WC* waist circumference, *TC* total cholesterol, *TG* triglyceride, *HDL* high density lipoprotein, *LDL* low density lipoprotein, *AST* aspartate aminotransferase, *ALT* alanine aminotransferase, *hs-CRP* high-sensitivity C-reactive protein, *MCP-1* monocyte chemoattractant protein-1, *TGFβ* transforming growth factor beta.

A significant p-values are indicated in bold (significance considered p < 0.05).

GLMS was performed to identify significant differences between median of PDI and CAV-1 rs3807992.

P* = with unadjusted (crude) model.

P** = with adjustments for potential confounding factors including (age, energy intake, physical activity education, job and marriage status).

We created an interaction model by using genotype categories and dietary intake groups (PDI, hPDI and UPDI) as fixed variables and confounding markers as covariates in GLMS.

GG genotype has 0 risk allele. AG genotype has one and AA genotype have two risk allele.

GG genotype is considered as a reference. Low adherence of PDI is considered as a reference.

**Table 6:** Interactions between CAV-1 rs3807992 and hPDI and uPDI on metabolic and inflammatory markers and anthropometric measures.

	hPDI			P**	uPDI			P**
	β (95%CI) (AA + AG) 1(Ref) GG				β (95%CI) (AA + AG) 1(Ref) GG			
	Crude	P*	Adjusted		Crude	P*	Adjusted	
WC (cm)	− 2.67 (− 7.12, 1.76)	0.23	− 1.6 (− 6.1, 2.72)	0.45	5.11 (0.69,9.53)	0.02	0.06 (< 0.001,0.12)	**0.04**
TC (mg/dl)	1.64 (− 16.53, 19.82)	0.85	10.34 (− 7.80, 28.49)	0.26	9.47(− 8.82, 27.78)	0.31	10.02 (− 8.54, 28.59)	0.29
HDL-C (mg/dl)	0.6 (− 4.83,6.04)	0.82	1.61(− 3.95, 7.17)	0.57	− 0.96 (− 6.43, 4.50)	0.72	− 1.1 (− 6.79, 4.59)	0.7
LDL-C (mg/dl)	3.45 (− 8.56, 15.48)	0.57	9.81 (− 2.1, 21.72)	0.1	− 3.39 (− 15.5, 8.71)	0.58	− 2.39 (− 14.65 , 9.85)	0.71
TC/HDL	− 0.14 (− 0.9, 0.61)	0.7	− 0.02 (− 0.82, 0.76)	0.94	0.68 (− 0.07,1.44)	0.07	0.72 (0.07,1.53)	**0.04**
ALT(IU L)	− 7.71 (− 14.35,− 1.06)	0.02	− 6.52 (− 13.48,− 0.43)	**0.04**	− 0.07 (− 3.90,3.74)	0.96	2.03 (− 5.12 , 9.18)	0.57
AST (IU L)	− 4.67 (− 8.40,− 0.94)	0.01	− 4.36 (− 8.27,− 0.44)	**0.02**	0.95 (− 5.79, 7.71)	0.78	− 0.41 (− 4.46, 3.65)	0.84
Insulin (pmol/L)	− 0.11 (− 0.23,0.003)	0.05	− 0.18 (− 0.28,− 0.03)	**0.03**	0.01 (− 0.1,0.12)	0.86	0.02 (− 0.09, 0.15)	0.65
hs-CRP (mg/L)	− 0.61 (− 3, 1.79)	0.62	− 0.67 (− 3.17, 1.82)	0.59	0.04 (− 2.38, 2.47)	0.97	0.17 (− 2.36, 2.72)	0.89
MCP-1(ng/ml)	− 30.79 (− 80.68, 19.09)	0.22	− 40.58 (− 93.15, 11.99)	0.13	55.69 (5.87,105.51)	0.02	0.37 (0.02,0.73)	**0.03**
TGFβ (ng/ml)	− 22.59 (40.80, − 3.39)	0.01	− 56.05 (− 78.96, − 3.14)	**0.001**	− 123.51 (− 331.45, 84.42)	0.24	− 192.52 (− 543.98, 158.93)	0.28
Galactin 3 (ng/ml)	2.56 (− 3.37,8.50)	0.39	2.14 (− 4.24, 8.53)	0.51	6.05 (0.02,12.09)	0.04	6.60 (0.26,12.94)	**0.04**

Values are represented as β (95%CI).

*WC* waist circumference, *TC* total cholesterol, *TG* triglyceride, *HDL* high density lipoprotein, *LDL* low density lipoprotein, *AST* aspartate aminotransferase, *ALT* alanine aminotransferase, *hs-CRP* high-sensitivity C-reactive protein, *MCP-1* monocyte chemoattractant protein-1, *TGFβ* transforming growth factor beta.

A significant p-values are indicated in bold (significance considered p < 0.05).

GLMS was performed to identify significant differences between median of PDI and CAV-1 rs3807992.

P* = with unadjusted (crude) model.

P** = with adjustments for potential confounding factors including (age, energy intake, physical activity education, job and marriage status).

We created an interaction model by using genotype categories and dietary intake groups (PDI, hPDI and uPDI) as fixed variables and confounding markers as covariates in GLMS.

GG genotype has 0 risk allele. AG genotype has one and AA genotype have two risk allele.

GG genotype is considered as a reference. Low adherence of hPDI and uPDI are considered as a reference.

### Interaction between PDI and *CAV-1 rs3807992* genotypes on metabolic and inflammatory markers and anthropometric measures

**Figure 1:**
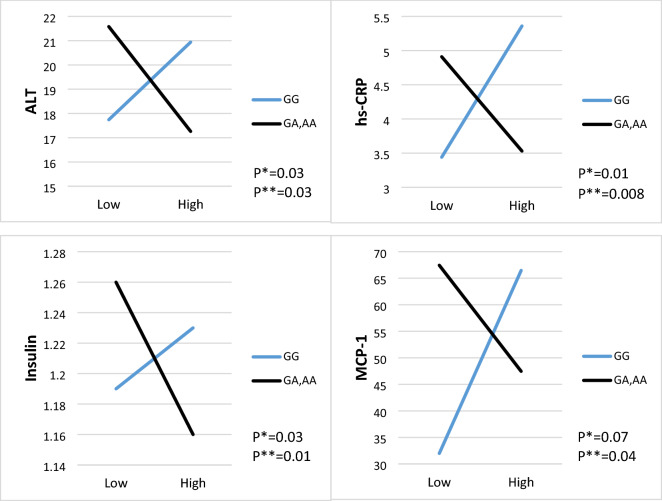
The interaction between CAV-1 SNP rs3807992 and plant-based diet index (PDI) on; (**a**) ALT, (**b**) hs-CRP, (**c**) insulin, (**d**) MCP-1. *P-value for curd (unadjusted) model. **P-value for the adjusted model by age, physical activity level, energy intake, education, job and marriage status. *ALT* alanine aminotransferase, *hs-CRP* high-sensitivity C-reactive protein, *MCP-1* monocyte chemoattractant protein-1.

There is a gene-diet interaction for PDI and CAV-1 polymorphism (rs3807992) on ALT (β: − 7.51, 95% CI − 14.45 to − 0.57, P: 0.03), hs-CRP (β: − 3.30, 95% CI − 5.76 to − 0.85, P: 0.008), insulin (β: − 0.14, 95% CI − 0.26 to − 0.02, P: 0.01), and MCP-1 (β: − 54.51, 95% CI − 107.72 to − 1.30, P: 0.04) in both crude and adjusted models (adjusting for age, energy intake, physical activity education, job and marriage status) (Table 5). Thus, the A-allele carriers who consumed higher PDI had lower ALT, insulin, MCP-1, and hs-CRP, compared to GG homozygotes (Fig. 1).

### Interaction between healthful PDI and *CAV-1 rs3807992* genotypes on metabolic and inflammatory markers and anthropometric measures

**Figure 2:**
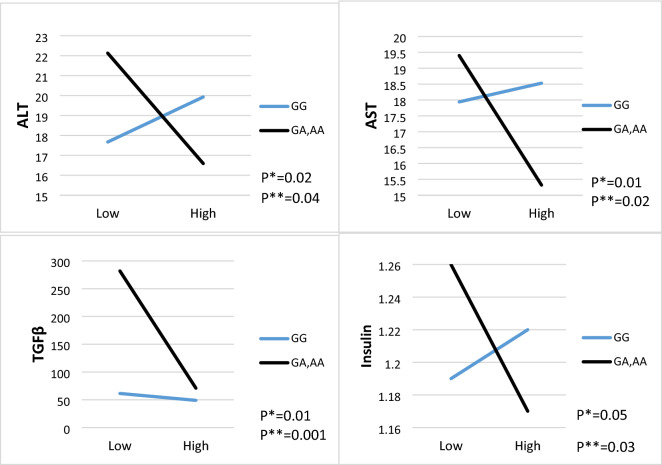
The interaction between CAV-1 SNP rs3807992 and healthy plant-based diet index (hPDI) on; (**a**) ALT, (**b**) AST, (**c**) TGF-β, (**d**) insulin. *P-value for curd (unadjusted) model. **P-value for the adjusted model by age, physical activity level, energy intake, education, job and marriage status. *AST* aspartate aminotransferase, *ALT* alanine aminotransferase, *TGFβ* transforming growth factor beta.

There were significant interactions between healthful PDI and rs3807992 on ALT (β: − 6.52, 95% CI − 13.48 to − 0.43, P: 0.04), AST (β: − 4.36, 95% CI − 8.27 to − 0.44, P: 0.02), insulin (β: − 0.18, 95% CI − 0.28 to − 0.03, P: 0.03), and TGF-b (β: − 56.05, 95% CI − 78.96 to − 3.14, P: 0.001) in both crude and adjusted adjustment model (Table 6). In particular, A-allele carriers were characterized by lower serum ALT, AST, insulin, and TGF-β, when following a healthful PDI, compared to GG homozygote. (Fig. 2).

### Interaction between unhealthful PDI and *CAV-1 rs3807992* genotypes on metabolic and inflammatory markers and anthropometric measures

**Figure 3:**
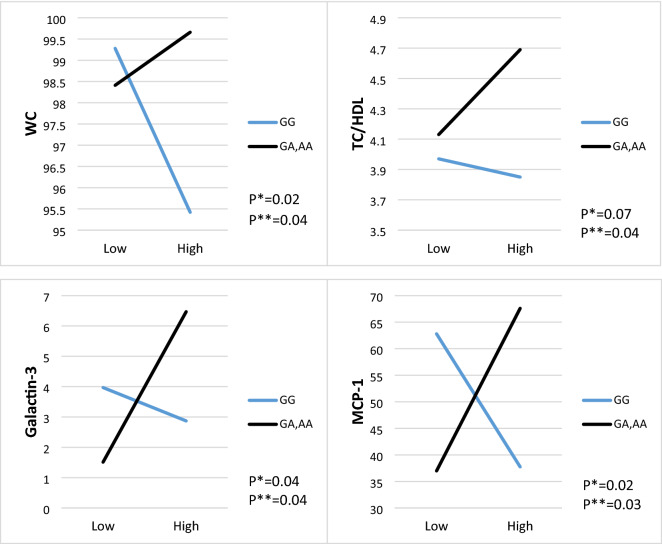
The interaction between CAV-1 SNP rs3807992 and Unhealthy plant-based diet index (uPDI) on; (**a**) WC, (**b**) TC/HDL, (**c**) Galactin-3, (**d**) MCP-1. *P-value for curd (unadjusted) model. **P-value for the adjusted model by age, physical activity level, energy intake, education, job and marriage status. *WC* waist circumference, *MCP-1* monocyte chemoattractant protein-1.

Risk-allele carriers who consumed higher unhealthful PDI had higher WC (β: 0.06, 95% CI 0.001–0.12, P: 0.04), TC/HDL (β: 0.72, 95% CI 0.07–1.53, P: 0.04), MCP-1(β: 0.37, 95% CI 0.02–0.73, P: 0.03), and galactin-3 (β: 6.60, 95% CI 0.26–12.94, P: 0.04). All data are shown in Table 6 and Fig. 3.

## Discussion

To our knowledge, this is the first study to have investigated the interactions between genetic variants of the CAV-1 gene with PDI on metabolic and inflammatory markers among Iranian women with overweight and obesity.

Our findings highlight a gene-diet interaction for PDI and CAV-1 polymorphism (rs3807992) on ALT, hs-CRP, insulin, and MCP-1 in both crude and adjusted models; such that the A- allele carriers who had higher PDI, had lower ALT, insulin, MCP-1, and hs-CRP compared to GG homozygotes. Based on our previous findings^46^, CAV-1 (rs3807992) might be related to incremented metabolic disease risk factors in women with overweight and obesity. We also previously posited that among minor allele carriers, insulin pathways may responsible for the relationship between CAV-1 rs3807992 and metabolic factors^46^.

In the present study, DBP, BFM, and FMI were higher in participants carrying the A allele compared to those in the GG genotype. There are some possible functional roles for CAV-1 may be inherited. It has been established by previous studies that CAV-1 has a major regulator function in fat distribution and genetic lipodystrophies in humans^47,48^. Furthermore, experimental studies have reported an association between CAV-1 mRNA expression in adipose tissues in obese women compared with lean subjects^49,50^. CAV-1- deficient mice had a smaller lean body phenotype than their wild-type counterparts^51,52^. Lipodystrophy has been observed in CAV-1 null mice due to a variety of functions attributed to caveolae in adipocytes, including lipid droplet dysfunction, adipocyte differentiation pathway disruption, abnormalities in cholesterol and fatty acid binding, transport, and storage, and an increase in insulin signaling^47,53^.

Moreover, A-allele carriers had significantly lower serum HDL and LDL compared to GG homozygotes. Additionally, there was no significant relationship between this polymorphism and other biochemical parameters containing TG, inflammatory markers (hs-CRP, MCP-1, PAI-1, and IL-1β), and liver enzymes in both crude and adjusted models. Concordant with our study, Khatibi et al. also found a significant association between CAV-1 genotypes with DBP^26^, and observed that participants with prevailing alleles had a lower risk of dyslipidemia, and that risk allele carriers, who adhered more to the Mediterranean‑DASH Intervention for Neurodegenerative Delay (MIND) diet, might present with lower dyslipidemia^26^.

In our study, we also observed a significant interaction between hPDI and rs3807992 on ALT, AST, insulin, and TGF-b in both crude and adjusted models. Indeed, A-allele carriers who had higher hPDI scores, had lower serum ALT, AST, insulin, and TGF-b compared to GG homozygote. Further, risk-allele carriers who had higher uPDI scores, had higher WC, TC/HDL, MCP-1, and galactin-3. No significant interactions were observed between CAV-1 rs3807992 variants and uPDI for other metabolic-related traits. A cross-sectional study in 2021 revealed that, among people following antioxidant-rich diets, ‘A’ allele carriers might be more vulnerable to cardiovascular disorders; the authors also reported no significant relationship between higher adherence to dietary total antioxidant capacity (DTAC) and changes in lipid profile, HOMA-IR, and body composition components^27^.

It is well accepted that the prevalence of obesity is directly related to dietary patterns^54^. PBD alleviates body fat due to the decreased caloric intake and incremented energy expenditure because of augmented thermogenesis^55^. Reduced use of saturated fats, which are usually derived from animal-based foods, might improve insulin sensitivity^55^; indeed, plant-based foods are also a main source of phytochemicals^56^. The consumption of phytochemicals, notably polyphenols, which exist in a variety of plant foods (e.g., berries, grapes, onions, apples, cacao, green tea, soy, whole grains, etc.), is related to reduced mortality and chronic disease risk^57–60^. The food compound of an uPDI might be associated with higher intakes of unpleasant nutrients and lower intakes of micronutrients and antioxidants, which can negatively affect metabolic syndrome and its factors. A high intake of simple carbohydrates from uPDI could also affect glucose control, lipid metabolism, and weight gain^61^, whilst decreased dietary fiber might impact glycemic control, insulin sensitivity, and augment inflammation. These effects could be related to reduced inflammation and oxidative stress^62,63^. Chronic inflammation is often evident in those with poor feeding habits and a sedentary lifestyle, features which are concomitantly associated with obesity progression^64,65^, in addition to various other pathologies, including asthma and Alzheimer disease, and different diseases related to unbalanced metabolisms such as T2DM, atherosclerosis, and cardiovascular diseases^66–69^. Metabolic inflammation consists of a complex mechanism containing crosstalk between different tissues (like adipose tissue and liver) through the whole body. Generally, this low-grade inflammation emerges when cellular stress is distinguished by the immune system^70^. Another mechanism posited to contribute to the progression of chronic inflammation involves the redundant storage of triglyceride (TG) lipids within adipose tissues. In murine models, exceeding TG storage in white adipose tissue (WAT) induces secretion of pro-inflammatory adipokines, such as IL-1, TNF-α, MCP-1, and IL-6, triggering systemic metabolic inflammation^71^.

Although the exact mechanism of the interaction between CAV-1 and diet has not yet been fully elucidated, some explanations have been proposed. Several studies have expressed that CAV-1 binds to endothelial nitric oxide synthase (eNOS) and HDL receptors in the caveolae and prevents their activity. Some diets, such as the PBD or anti-inflammatory diets, can dislocate CAV-1 from caveolae to the cytoplasm, leading to a reduction in the CAV-1 level, ameliorating the prohibitory effects on HDL and eNOS receptors^72^. Decreased nitric oxide (NO) generation, as a result of increased CAV-1 expression, has been thought to occur from prolonged exposure to high glucose, may play a vital role in inflammatory pathways and expansion of inflammation^73^. Hence, it is not surprising that higher adherence to a uPDI can augment MCP-1 by changing the expression of CAV-1 and other genes.

Diet and plasma-derived nutrients may influence metabolic markers by interacting with caveolae-associated cellular signaling, according to new research^36^. In this regard, Oberleithner et al. suggest that serum sodium and potassium can control eNOS binding to the caveolae membrane and activity^74^. As a result, the beneficial benefits of a healthy PDI might be attributed to components like vegetables and fruits, which have a greater impact on potassium and sodium balance.

Beside, the lower liver enzyme in A-allele carriers following an hPDI in the present study can be dependent on the caffeine intake during the hPDI. In this term, we previously found a decreased AST in A-allele carriers who consumed more caffeine in a healthy dietary pattern^45^.Interestingly, a candidate gene for the favorable impact of caffeine consumption is located near the CAV-1 gene^75^, Therefor, we suggested a possible interaction between caffeine and CAV-1, which we hope will be confirmed in future study.

In the current study, we observed a relationship between CAV-1 with body composition, which could suggest a CAV-1 lipogenic pathway interaction. The expression of CAV-1 is found in most normal organs but notably assuming that CAV-1 is greatly expressed in adipose tissue^76^ and the interplay between CAV-1 gene and lipogenic genes has been expressed^77^. Visceral fat could also be associated with changes in circulating fatty acid composition^78^. CAV‐1 interacts with two popular receptors that augment oxidative stress: the angiotensin II receptor and mineralocorticoid receptor (MR)^79^. MR blockade has been shown to reduce NADPH Oxidase 4 (NOX4) expression and lower reactive oxygen species in the kidney and heart^80,81^.

Several studies have revealed that CAV-1 is associated with oxidative stress, as reactive species could impact the expression, degradation, post-translational changes, and trafficking of Caveolae membranes^82^. Hence, because of the impact of the CAV-1 gene with oxidative stress, it seems that adherence to hPDI could be capable of lowering the metabolic markers, and, as a result, reducing the likelihood of metabolic diseases in women with obesity carrying a risk the allele of ‘A’. Indeed, the current study provides some support for the consideration of particular dietary guidelines based on genotypes.

Although we provide novel findings of gene-diet interactions, some limitations should be considered in the interpretation of the study. First, the results of this cross-sectional study, although nationally illustrative, cannot indicate a causal relationship. Second, even though we applied a validated FFQ, measurement errors are possible. Third, our study only included women, therefore, results are not generalizable to men. Finally, although we considered potential confounders, residual confounding may still exist. We did not have any data related to family history of cancer; however, the expression of CAV-1 can be effected by this cofounding marker.

## Conclusion

In conclusion, the results of the current study suggest that diet, gene variants, and their interaction, should be considered in metabolic diseases risk assessment. PDIs may provide advantages in the prohibition of chronic disease. In future studies, investigating samples from a greater geographic area with wide ranges of ages in both sexes and larger sample sizes might prove more important findings. This work clearly highlights the value of considering genotypes in dietary planning and modification. Therefore, these findings from nutrigenetic studies in future may be combine with a patient's genetic history to give more relevant and customized nutritional recommendations for women with obesity in order to avoid or reduce cardiovascular disease.

## Data Availability

The data are not publicly available due to containing private information of participants. Data are however available from the authors upon reasonable request and with permission of Khadijeh Mirzaei.

## Abbreviations

AST: Aspartate aminotransferase
BIA: Bioelectrical impedance analysis
BMI: Body mass index
bp: Base pair
BFM: Body fat mass
CRP: C-reactive protein
CVD: Cardiovascular disease
DBP: Diastolic blood pressure
DNA: Deoxyribonucleic acid
DP: Dietary pattern
eNOS: Endothelial nitric oxide synthase
FMI: Fat mass index
FFQ: Food frequency questionnaire
GLM: General linear model
GWAS: Genome-wide association studies
HDL: High-density lipoprotein cholesterol
IR: Insulin Receptor
hs-CRP: High sensitivity C-reactive protein
IPAQ: International physical activity questionnaire
LDL: Low-density lipoprotein
MA: Minor allele
PBD: Plant-based diet
PDI: Plant-based dietary index
PCR: Polymerase chain reaction
PDI: Plant-based dietary index
RFLP: Restriction fragment length polymorphism
SNP: Single nucleotide polymorphism
TC: Total cholesterol
TG: Triglyceride
WC: Waist circumference
WHO: World Health Organization
